# Comparison of the efficacy of low-molecular-weight heparin and fondaparinux sodium after total knee arthroplasty: a retrospective cohort study

**DOI:** 10.1186/s12891-023-06674-6

**Published:** 2023-07-04

**Authors:** Tianxiang Yang, Zige Liu, Bowen Zhang, Jinning Zhang, Anning Ma, Dede Cao, Desheng Chen

**Affiliations:** 1 Department of Clinical Medicine, Ningxia Medical University, Yinchuan, 750004 China; 2 Department of Clinical Medicine, Guangxi Medical University, Nanning, 530021 China; 3 Department of Orthopedics, People’s Hospital of Ningxia Hui Autonomous Region, Yinchuan, 750002 China

**Keywords:** Knee osteoarthritis, Total knee arthroplasty, Low-molecular-weight heparin, Fondaparinux sodium, Coagulation indicator

## Abstract

**Background:**

Low-molecular-weight heparin (LMWH) and fondaparinux sodium (FPX) are routinely used to prevent deep vein thrombosis (DVT) after total knee arthroplasty (TKA). In this study, we compared the effects of these agents in preventing post-TKA DVT.

**Methods:**

Clinical data of patients who underwent unilateral TKA for unicompartmental knee osteoarthritis at the Ningxia Medical University General Hospital between September 2021 and June 2022 were retrospectively analyzed. Based on the anticoagulation agent used, the patients were divided into LMWH and FPX groups (34 and 37 patients, respectively). Changes in perioperative coagulation-related indicators, d-dimer and platelet count, perioperative complete blood count, amount of blood loss, lower-limb DVT, pulmonary embolism, and allogeneic blood transfusion were determined.

**Results:**

Intergroup differences in d-dimer or fibrinogen (FBG) levels before and 1 or 3 days after surgery were not significant (all *p* > 0.05); within-group pairwise comparisons indicated significant differences (all, *p* < 0.05). Intergroup differences in preoperative prothrombin time (PT), thrombin time, activated partial PT, and international normalized ratio were not significant (all *p* > 0.05), whereas significant differences were detected on postoperative days 1 and 3 (all *p* < 0.05). Intergroup differences in platelet counts before and 1 or 3 days after surgery were not significantly different (all *p* > 0.05). Pairwise comparisons of hemoglobin and hematocrit levels between patients in the same group before and 1 or 3 days after surgery revealed significant differences in both groups (all *p* < 0.05); however, intergroup differences were not significant (all *p* > 0.05). Although intergroup differences in visual analog scale (VAS) scores before and 1 or 3 days after surgery were not significant (*p* > 0.05), we detected significant intragroup differences in VAS scores before and 1 or 3 days after surgery (*p* < 0.05). The treatment cost ratio was significantly lower in the LMWH group than in the FPX group (*p* < 0.05).

**Conclusion:**

Both LMWH and FPX can effectively prevent DVT after TKA. There are some suggestive signals that FPX may have more beneficial pharmacological effects and clinical significance, while LMWH is cheaper and therefore more economical.

## Background

Knee osteoarthritis (KOA) is a common clinical joint disease that mainly develops in the elderly. It is a chronic degenerative disease with a high incidence of disability and deformity. The affected patients experience continuous knee swelling and pain, which severely affects their physical and mental health [1, 2]. The incidence of KOA in China has been reported to be 18%, [3, 4] with over 80 million people developing KOA each year, of whom approximately 20,000 develop advanced KOA. With the aging of the Chinese population, the familial and social burdens of KOA are progressively increasing. Although total knee arthroplasty (TKA) is an important treatment option for patients with severe or disabling KOA, [5] deep vein thrombosis (DVT), one of the most common complications after TKA, has a considerable bearing on the safety of the procedure. The incidence of DVT after TKA in patients without preventive drug treatment is as high as 58.1% [6]. Accordingly, the use of appropriate anticoagulant drugs to prevent postoperative thrombosis is vital for improving the prognosis of patients. Low-molecular-weight heparin (LMWH) and fondaparinux sodium (FPX) are commonly used to prevent DVT after TKA. However, data on the effects of both after TKA are still lacking. In this study, we compared the effects of LMWH and FPX in preventing lower-limb DVT after TKA. Our findings may serve as a reference for the selection of appropriate anticoagulant treatments after TKA.

## Methods

### General characteristics

Clinical data of 71 patients who underwent unilateral TKA for KOA at the Ningxia Medical University General Hospital between September 2021 and June 2022 were collected and analyzed. The patients were divided into LMWH and FPX groups (34 and 37 patients, respectively) based on the agent used for postoperative anticoagulation. An assessment of general preoperative characteristics, including age, height, weight, body mass index (BMI), sex, surgical procedure, surgical site, complications, disease duration, blood pressure, and pulse pressure, revealed that differences between the two groups were not significant (all, *p* > 0.05) (Table 1).

**Table 1:** Baseline characteristics of the two groups of patients

Group		LMWH group (n = 34)	FPX group (n = 37)
Age (years)		66.15 ± 6.82	65.84 ± 5.12
Height (m)		1.60 ± 0.07	1.61 ± 0.07
Weight (kg)		69.25 ± 11.76	65.00(11.60)
BMI (kg/m^2^)		26.83 ± 3.74	25.33 ± 3.21
Sex (%)	M	4(11.76)	10(27.03)
	F	30(88.24)	27(72.97)
Surgical site (%)	L	18(52.94)	17(45.95)
	R	16(47.06)	20(54.05)
Comorbidities	Hypertension	19(55.88)	22(59.46)
	Diabetes	12(35.29)	7(18.92)
Disease duration (months)		96.00(84.00)	120.00(51.00)
Preoperative systolic pressure (mmHg, 1 mmHg = 0.133 kPa)		132.24 ± 16.18	131.97 ± 13.12
Preoperative diastolic pressure (mmHg)		79.97 ± 10.98	78.50(13.00)
Pulse pressure (mmHg)		52.26 ± 12.26	50.81 ± 9.27

### Inclusion criteria

The following patients were included: (1) patients who met the diagnostic criteria of the Guidelines for the Diagnosis and Treatment of Osteoarthritis in China (2021 edition); [7] (2) patients diagnosed with KOA based on imaging findings; (3) patients whose symptoms did not markedly improve after conservative treatment and who were willing to undergo joint surgery; (4) patients who underwent unilateral surgical treatment; (5) patients without lower-limb abnormalities on preoperative color Doppler ultrasound examination; and (6) patients who provided written informed consent or whose family members provided such consent. The exclusion criteria were as follows: [8] (1) severe hematologic disease; (2) an abnormal preoperative blood count or long-term anticoagulant use; (3) patients with a history of thrombosis and lower extremity vascular disease; (4) concomitant tuberculosis, cancer, or other serious systemic diseases; (5) severe knee deformities (flexion contracture deformity > 20 °, varus deformity > 25 ° or valgus deformity > 15 °, severe collateral ligament contracture deformity or rupture, and severe bone defects around the knee joint); and (6) incomplete clinical examination and test data. The study was approved by the Institutional Review Board of the General Hospital of Ningxia Medical University (IRB no.: 2020 − 974).

### Methods

All patients underwent elective surgery after routine preoperative tests and examinations. The prosthesis model was initially selected based on physical examination and imaging results. To prevent infection, antibiotics were administered intravenously 30 min prior to the operation. All patients were placed in the supine position, administered general anesthesia, and underwent routine disinfection and draping. An aseptic film was used to protect the skin. Blood flow to the affected limb was occluded using a tourniquet, and a pneumatic tourniquet at a pressure of 260 mmHg was applied for hemostasis.

#### Surgical procedure

 All patients were operated on by the same group of surgeons according to the same surgical standards. A midline incision was made in the knee, and routine surgery was performed. After prosthesis fixation and routine hemostasis, a drainage tube was inserted, the incision was sutured in a flexed knee position, and the wound was covered with a sterile dressing and compression bandage. The amount of intraoperative blood loss was recorded, and no blood transfusion was required in either group. For both groups, surgery was completed in the absence of any adverse events.

#### Postoperative anticoagulation

The choice of anticoagulant drugs was jointly decided by the physician and patient following consultation. The physician explained the advantages and disadvantages of the two drugs to the patient, and presented their preferred option based on the condition and financial status of the patient. The selected agent was then administered after receiving patient consent. The timing and dosage of anticoagulants were selected according to the “Guidelines for the Prevention of Major Orthopedic Venous Thromboembolism (2016 Edition).” [9]. The LMWH group received low molecular weight heparin calcium injection subcutaneously at half the normal dose 4–6 h after the procedure. The normal dose was injected (one injection per day) on the following day. The FPX group was subcutaneously injected with a 2.5 mg dose, starting at 6 h after surgery (one injection per day). The treatment duration was 2 weeks.

#### Other postoperative management

All patients were administered antibiotics to prevent postoperative infection. The drainage tube was removed on postoperative day 2, and the volume of postoperative drainage fluid was recorded. Regarding postoperative functional exercises, all patients moved to the ground on the first day after surgery with the help of a walking aid; ankle pump exercises and quadriceps training were performed on postoperative day 1, followed by leg press exercises on postoperative day 2. Straight leg raising, leg bending, and active knee flexion and extension exercises were performed on postoperative day 3. Routine color Doppler ultrasound examination of the deep veins of both lower limbs was performed on postoperative days 2, 3, and 14, and pulmonary angiography was performed if necessary. Discharge criteria: The patient had no severe anemia, poor wound healing, and other complications after operation. Knee joint pain was tolerable and joint flexion could reach 90 °.

### Observational indices

#### Perioperative coagulation-related indices

Plasma D-dimer, activated partial prothrombin time (APTT), prothrombin time (PT), thrombin time (TT), fibrinogen (FBG), platelet (PLT) count, and international normalized ratio (INR) were determined preoperatively and on postoperative days 1 and 3.

#### Perioperative blood loss-related indices

Hematocrit (Hct) and hemoglobin (Hb) levels were measured preoperatively and on postoperative days 1 and 3. Intraoperative blood loss, volume of postoperative drainage fluid, apparent blood loss, hidden blood loss, and total blood loss were recorded for both groups. The Nadler equation was used to calculate the total blood volume of patients. The Gross formula was used to calculate blood loss, and the total blood volume calculation formula was (1) k1 × h^3^ + k2 × m + k3 (where h is height, unit: m, m is weight, unit: kg); k values were as follows: males- k1 = 0.366 9, k2 = 0.032 19, k3 = 0.604 1 and females- k1 = 0.356 1, k2 = 0.033 08, k3 = 0.183 3. (2) Lost red blood cell volume = patient blood volume × (hematocrit before operation - hematocrit 3 days after operation); Total blood loss after operation = lost red blood cell volume/hematocrit before operation; Total perioperative blood loss = total postoperative blood loss + allogeneic blood transfusion; Hidden blood loss = total blood loss during perioperative period- visible blood loss; Visible blood loss = drainage bottle drainage volume during operation- physiological saline volume during operation + increased net weight/blood density of gauze during operation + drainage volume in drainage bag after operation. (3) Transfusion patients received 1U red blood cell suspension equal to 200 mL of standard red blood cell volume.

#### Other perioperative indices

Preoperative and postoperative visual analog scale (VAS) pain scores, operation time, postoperative blood transfusion, postoperative DVT, pulmonary embolism, and cost ratio of anticoagulant treatment (treatment cost/total efficacy) [5] were recorded for both groups.

### Statistical analysis

The clinical related information of patients in this study was collected through our HIS electronic medical record system and ward physical examination. Data collection was conducted under the guidance and supervision of clinical and medical record room doctors, with unified data collection methods. On the day of data collection, a dedicated person was responsible for verifying the database and promptly supplementing and correcting it. All clinical data were analyzed using SPSS 25.0 statistical software. Measurement data of normal distribution are expressed as the means ± standard deviation, the measurement data of non normal distribution are expressed as median and quartile spacing [M (P25-P75)], and the inter group comparison was performed by independent t-test or nonparametric test. The counting data are expressed as percentage (%), the χ^2^ test was used for comparisons of count data between the groups. The level of significance was set at α = 0.05.

## Results

### Between-group comparisons of perioperative coagulation-related indices

There were no significant intergroup differences in d-dimer or FBG levels preoperatively or on postoperative days 1 and 3 (all *p* > 0.05). However, pairwise comparisons within the groups indicated significant differences (all *p* < 0.05). Furthermore, there were no significant intergroup differences in preoperative PT, TT, APTT, or INR (all *p* > 0.05). However, on postoperative days 1 and 3, the intergroup differences in these parameters were significant (all *p* < 0.05). There were no significant differences between the two groups with respect to PLT count, either preoperatively or on postoperative days 1 or 3 (all *p* > 0.05) (Table 2).

**Table 2:** Perioperative coagulation-related indicators in the two groups of patients

Group		LMWH group (n = 34)	FPX group (n = 37)	*t/Z*	*p*
d-dimer (mg/L)	Preoperative	0.33(0.45)	0.44(0.37)	-1.036	0.300
	Postoperative day 1	3.90(5.30)^*^	5.91(4.91)	-1.727	0.084
	Postoperative day 3	1.64(1.56)^*#^	1.58(0.84)	-0.679	0.497
FBG (g/L)	Preoperative	2.61(0.69)	2.94 ± 0.60	-1.934	0.053
	Postoperative day 1	3.09(1.04)^*^	3.29 ± 0.51^*^	-1.335	0.182
	Postoperative day 3	4.84 ± 1.26^*#^	4.91 ± 0.59^*#^	−0.303	0.763
PT (s)	Preoperative	10.67 ± 0.48	10.79 ± 0.46	−1.102	0.274
	Postoperative day 1	11.15(1.20)^*^	12.50(1.20)	-5.279	0.000
	Postoperative day 3	10.95(0.80)^*#^	11.50(1.30)	-3.473	0.001
TT (s)	Preoperative	14.96 ± 1.02	14.90 ± 1.04	0.251	0.802
	Postoperative day 1	17.28 ± 1.89^*^	13.92 ± 1.07^*^	9.115	0.000
	Postoperative day 3	15.56 ± 1.93^#^	13.45(0.70)	-4.630	0.000
APTT (s)	Preoperative	29.20(5.40)	29.95 ± 3.49	-0.714	0.475
	Postoperative day 1	26.30(3.80)^*^	32.39 ± 4.06^*^	-5.025	0.000
	Postoperative day 3	26.75(4.20)^*^	29.90(4.30)	-4.202	0.000
INR	Preoperative	0.94(0.11)	1.00 ± 0.06	-1.672	0.95
	Postoperative day 1	0.97(0.14)	1.13(0.11)	-5.675	0.000
	Postoperative day 3	0.98(0.07)^#^	1.04(1.12)	-5.068	0.000
PLT count	Preoperative	226.60 ± 65.28	253.68 ± 76.06	−1.603	0.114
	Postoperative day 1	202.68 ± 54.72^*^	227.30 ± 77.83^*^	−1.529	0.131
	Postoperative day 3	174.50(78.00)^*^	203.57 ± 69.19^*#^	-1.145	0.252

Note: ^*^p < 0.05 compared with the preoperative values; ^#^*p* < 0.05 compared with the values on postoperative day 1; LMWH: Low-molecular-weight heparin; FPX: fondaparinux sodium; FBG: fibrinogen; PT: prothrombin time; TT: thrombin time; APTT: activated partial prothrombin time; INR: international normalized ratio; PLT: platelet

### Between-group comparisons of perioperative blood loss-related indices

Pairwise comparisons of Hb and Hct levels between patients in the same group preoperatively or on postoperative days 1 or 3 between the two groups revealed that the differences were significant (all *p* < 0.05), whereas intergroup differences were non-significant (all *p* > 0.05). Furthermore, in intergroup comparisons, we detected no significant differences in intraoperative bleeding, volume of postoperative drainage fluid, apparent blood loss, hidden blood loss, or total blood loss (all *p* > 0.05) (Table 3).

**Table 3:** Perioperative blood loss-related indicators of the two groups of patients

Group		LMWH group (n = 34)	FPX group (n = 37)	*t/Z*	*p*
Hb (g/L)	Preoperative	135.50 ± 12.45	138.50(12.00)	-1.377	0.168
	Postoperative day 1	122.18 ± 13.30^*^	126.24 ± 11.41^*^	−1.386	0.170
	Postoperative day 3	100.50(20.00)	104.92 ± 18.49^*#^	-0.472	0.637
Hct (%)	Preoperative	40.45(5.70)	42.08 ± 3.57	-1.520	0.129
	Postoperative day 1	36.25 ± 3.88^*^	37.66 ± 3.08^*^	−1.706	0.093
	Postoperative day 3	30.83 ± 4.30^*#^	31.02 ± 4.90^*#^	−0.167	0.867
Intraoperative blood loss (mL)		100.00(70.00)	90.00(74.00)	-1.419	0.156
Volume of postoperative drainage fluid (mL)		70.00(70)	100.00(135.00)	-1.379	0.168
Visible blood loss (mL)		172.35 ± 91.38	167.50(150.00)	-0.386	0.700
Hidden blood loss (mL)		828.09 ± 289.24	900.29 ± 445.88	−0.816	0.418
Total blood loss (mL)		1000.44 ± 268.36	1095.43 ± 503.43	−1.003	0.320

Note: ^*^p < 0.05 compared with the preoperative values; ^#^*p* < 0.05 compared with the values on postoperative day 1; LMWH Low-molecular-weight heparin; FPX fondaparinux sodium; Hb hemoglobin; Hct Hematocrit

### Between-group comparisons of other perioperative indices

We detected no significant intergroup differences in VAS scores either preoperatively and on postoperative days 1 and 3 (*p* > 0.05). Nevertheless, there were significant differences between preoperative VAS scores and scores on postoperative days 1 and 3 (*p* < 0.05). There were no significant differences between the groups with respect to the operation time, postoperative transfusion rate, and DVT incidence (*p* > 0.05). One patient with DVT in the LMWH group developed limb swelling, pain, and numbness, which resolved after regular treatment. The cost of a LMWH injection was 24.26 CNY per injection, with a total regimen cost of 339.64 CNY. The total efficiency for DVT prevention was 85.29% (29/34), and the treatment cost ratio was 3.98. Comparatively, the cost of FPX was 125.00 CNY per injection, with a total regimen cost of 1750 CNY. The total efficiency for DVT prevention was 91.89% (34/37), and the treatment cost ratio was 19.04. Thus, the treatment cost ratio was lower in the LMWH group than in the FPX group and the intergroup difference in this regard was significant (*p* < 0.05). There were no cases of pulmonary embolism (Table 4).

**Table 4:** Other perioperative indicators of the two groups of patients

Group		LMWH group (n = 34)	FPX group (n = 37)	*Z*/χ^2^	*p*
VAS score	Preoperative	3.00(0.00)	3.00(0.00)	-0.493	0.622
	Postoperative day 1	4.00(1.00)	4.50(1.00)	-0.689	0.491
	Postoperative day 3	5.00(1.25)	4.00(1.75)	-0.399	0.690
Operation time (min)		91.21 ± 7.21	88.00(9.00)	-0.463	0.643
Postoperative blood transfusion		1	4	1.676	0.195
DVT		5	3	0.771	0.380
Treatment cost ratio		3.98	19.04	11.134	0.001

Note: ^*^p < 0.05 compared with the preoperative values; VAS visual analog scale; DVT deep vein thrombosis

## Discussion

KOA is a chronic degenerative joint disease characterized by articular cartilage lesions and bone hyperplasia that is common in the elderly. Long-term knee pain and reduced function severely affect the daily activities and physical and mental health of the affected patients [10, 11]. Clinically, although TKA is often performed for some patients with severe clinical symptoms and loss of joint function, there is a very high risk of developing DVT after TKA, [12] and severe disease may result in venous insufficiency and concurrent life-threatening pulmonary embolism. The main factors contributing to the development of DVT include [13] venous vessel wall injury, slow venous blood flow, and a hypercoagulable state. The use of anesthetics leads to a slowing of systemic blood flow, and postoperative bed rest affects coagulation function [14, 15]. Therefore, postoperative anticoagulant interventions have become essential for preventing DVT after TKA, among which LMWH and FPX are the most widely used clinically.

LMWH, the most commonly used anticoagulant and antithrombotic, effectively inhibits thrombin activation, promotes blood flow, and ensures the normal flow of blood, thereby significantly reducing the incidence of DVT after major orthopedic surgery without increasing the risk of substantial bleeding [16, 17]. The dosage can be adjusted depending on body weight, making its use relatively safe. Although LMWH is rarely associated with severe bleeding complications, there remains a small probability of heparin-induced thrombocytopenia [9]. FPX is a synthetic selective inhibitor of activated factor X (factor Xa). The simple pentose structure of FPX significantly enhances its affinity for antithrombin and it specifically binds to the active site of antithrombin via non-covalent bonding, resulting in a rapid inhibition of factor Xa, thereby reducing thrombin production and fibrin formation. Unlike LMWH, FPX is not predicted to bind to platelet factor IV and does not cross-react with the plasma of patients with heparin-induced thrombocytopenia. It has unique anticoagulant activity and a long half-life (10–15 h), and significantly reduces the risk of bleeding and other adverse effects. Moreover, it has good selectivity, and vasoconstriction, fibrin synthesis, and platelet aggregation can be attenuated by controlling the treatment duration. When used in preventive treatment, FPX has a stable metabolic environment that has little effect on its blood concentration. However, it is contraindicated in patients with severe renal insufficiency and creatinine clearance of < 20 mL/min, [9, 18, 19] both conditions of which generally do not require routine hematological monitoring.

FBG is a hepatocyte-synthesized protein with coagulation function. Its structure, which has mirror symmetry, is cleaved into active peptides upon the initiation of hemostasis, forming fibrin monomers, which subsequently polymerize to form a γ-fibrin fibrillar network [20]. Following hemostasis, the healing process is initiated, the first stage of which is fibrinolysis. In this process, fibrin is degraded by the action of enzymes to form a d-dimer, the plasma levels of which are indicative of coagulation and fibrinolytic status [21, 22]. In the present study, differences between the LMWH and FPX groups with respect to coagulation were primarily in terms of APTT and TT, with the normal range of APTT, a sensitive indicator of endogenous coagulation, being 23–36 s. Prolonged APTT is often observed in cases with reduced levels of coagulation factors II, V, and VII or FIB deficiency, and a shortened APTT is considered to be indicative of a hypercoagulable state [23]. The normal range of TT, which represents the common pathway of coagulation and reflects the inhibition of fibrin conversion, is 9–21 s, and a prolonged TT is generally associated with reduced levels of FIB and hyperactive fibrinolysis [24, 25]. Herein, we found that in the LMWH group, APTT was lower, and TT was higher than the corresponding preoperative times, whereas the opposite was observed in the FPX group. These trends suggest that patients in the LMWH group may have higher coagulation and fibrinolytic states than those in the FPX group, leading to suggestive results: in pharmacology, FPX may be more beneficial and clinically meaningful.

Perioperative blood loss is a major short-term complication of knee arthroplasty, with blood loss generally ranging from 300 to 2400 mL [26]. Excessive perioperative blood loss increases the surgical risk of arthroplasty and the probability of other postoperative complications [27]. In the present study, we detected no significant differences between the two groups with respect to intraoperative blood loss, volume of postoperative drainage fluid, visible blood loss, hidden blood loss, or total blood loss, and similarly, intergroup differences in postoperative VAS scores, operation time, postoperative transfusion rate, and incidence of DVT were non-significant. These findings indicate that in terms of postoperative pain, perioperative blood loss, and incidence of DVT, there were no significant differences in the use of LMWH and FPX for the prevention of DVT after TKA. However, given that the cost of treatment was lower in the LMWH group than in the FPX group, the use of LMWH for DVT prevention can be considered more economical.

## Conclusions

Both LMWH and FPX were demonstrated to be effective in preventing DVT after TKA, and there were no significant differences between these two agents with respect to postoperative pain, perioperative blood loss, or incidence of DVT. However, we have also drawn some suggestive signals that the pharmacological effects of FPX may be considered more beneficial and more clinically significant, whereas the cost of LMWH in preventing DVT is lower and thus its usage is more economical. The limitations of this study are as follows: (1) As this was a single center study, it involved a single research population and a small sample size; (2) Because this was a retrospective cohort study, some data were missing which may have affected the results, thus the statistical analysis lacks sufficient assurance; (3) Due to the relatively low incidence of VTE and the difficulty in significantly expanding the study sample size, subgroup analysis was lacking; (4) We acknowledge that this was a convenient sample and there was no prior sample size to determine sufficient queue size. We intended that the data would offer indicative efficacy signals and not a definitive assessment. In the future, it is necessary to expand the sample size to verify the accuracy of the results.

## Data Availability

The datasets used during the current study are available from the corresponding author on reasonable request.

## Declarations

### Author information

Tianxiang Yang: Master’s degree; Research interests: Diagnosis and treatment of joint diseases.

Desheng Chen: MD, MSc; Professor/Chief Physician; Research interests: Clinical and basic research in joint surgery.

## Abbreviations

APTT: Activated partial prothrombin time
BMI: Body mass index
DVT: Deep vein thrombosis
FBG: Fibrinogen
FPX: Fondaparinux sodium
Hb: Hemoglobin
Hct: Hematocrit
INR: International normalized ratio
KOA: Knee osteoarthritis
LMWH: Low-molecular-weight heparin
PLT: Platelets
PT: Prothrombin time
TKA: Total knee arthroplasty
TT: Thrombin time
VAS: Visual analog scale

## References

[1] Han Y, Huang H, Pan J (2019). Meta-analysis comparing platelet-rich plasma vs hyaluronic acid injection in patients with knee osteoarthritis. Pain Med.

[2] Wallace IJ, Worthington S, Felson DT (2017). Knee osteoarthritis has doubled in prevalence since the mid-20th century. Proc Natl Acad Sci USA.

[3] Wang B, Xing D, Dong SJ (2018). Prevalence and disease burden of knee osteoarthritis in China: a systematic review. Chin J Evid-Based Med.

[4] Zhang X, Ma D, Pan J (2021). Effects of different applications of tranexamic acid on perioperative blood transfusion rate and postoperative pain in unilateral total knee arthroplasty. Adv Ther.

[5] Yang XN, Zhao LH, Huang J (2021). Comparative study on Enoxaparin Sodium and Fondaparinux Sodium in the Prevention of deep venous thrombosis after knee arthroplasty. Evaluation and analysis of drug-use in hospitals of China.

[6] Wu Q, Zou SY, Liu LF (2022). Analysis of related factors in the distribution of lower limb thrombosis after total knee arthroplasty. Orthop J China.

[7] The Joint Surgery Branch of the Chinese Orthopaedic Association; The Subspecialty Group of Osteoarthritis, Chinese Association of Orthopaedic Surgeons; The National Clinical Research Center for Geriatric Disorders (2021). Chinese guideline for diagnosis and treatment of osteoarthritis (2021 edition). Chin J Orthop.

[8] Jiang J, Xu JJ, Yu BC (2021). Comparison of clinical efficacy between unicondylar arthroplasty and total knee arthroplasty for knee osteoarthritis. Contemp Med.

[9] Tian W (2016). Guidelines for prevention of venous thromboembolism in major orthopedic surgery in china. Chin J Orthop.

[10] Blevins JL, Chiu YF, Lyman S (2019). Comparison of expectations and outcomes in rheumatoid arthritis versus osteoarthritis patients undergoing total knee arthroplasty. J Arthroplasty.

[11] Naylor JM, Pocovi N, Descallar J (2019). Participation in regular physical activity after total knee or hip arthroplasty for osteoarthritis: prevalence, associated factors, and type. Arthritis Care Res (Hoboken).

[12] Zhang JL, Feng H, Tang XY. Clinical progress of integrated traditional chinese and western medicine for the prevention and treatment of lower extremity deep vein thrombosis after orthopedic surgery. Volume 34. JCM; 2022. pp. 189–94.

[13] Zhang C, Song GR, Liu ZG (2020). Comparative analysis of the efficacy and safety of low molecular weight heparin and rivaroxaban in prevention of venous thrombosis after TKA. Shaanxi Med J.

[14] Gao Y, Qin H, Fan GF (2019). Risk factors for venous thromboembolism in patients with craniocerebral trauma. J Chin Pract Diagn Ther.

[15] Bui MH, Hung DD, Vinh PQ (2019). Frequency and risk factor of lower-limb deep vein thrombosis after major orthopedic surgery in vietnamese patients. Open Access Maced J Med Sci.

[16] Gwam CU, George NE, Etcheson JI (2019). Cementless versus cemented fixation in total knee arthroplasty: usage, costs, and complications during the inpatient period. J Knee Surg.

[17] Geerts WH, Bergqvist D, Pineo GF (2008). Prevention of venous thromboembolism: american college of chest Physicians Evidence⁃Based clinical practice guidelines (8th Edition). Chest.

[18] The expert group for the compilation. of “Expert Consensus on the Pharmaceutical Practice of Fondaparinux Sodium” by the Clinical Pharmacy Branch of the Chinese Medical Association, Expert consensus on pharmaceutical practice of fondaparinux sodium [J/OL]. Her Med:1–22.

[19] Zhang XY, Wang LJ (2018). Clinical study on Danhong Injection combined with fondaparinux sodium in treatment of pulmonary embolism. Drugs & Clinic.

[20] Battaglia S, Maiola V, Savastio G (2017). Osteomyocutaneous fibular flap harvesting: computer-assisted planning of perforator vessels using computed Tomographic Angiography scan and cutting guide. J Craniomaxillofac Surg.

[21] Ren K, Wu C (2022). Relationship between Perioperative Coagulation related indexes and flap complications after Limb Trauma Free Flap Repair. Chin J Thromb Hemostasis.

[22] Liu HB, Lu P, Li CM (2020). Value of several coagulation indexes inpatients with nephrotic syndrome. Shaanxi Med J.

[23] Alasiry E, Benyamin AF, Saleh S (2020). Correlation of wells score, prothrombin time, activated partial thromboplastin time, fibrinogen and D-Dimer levels with doppler ultrasonography in suspected deep vein thrombosis patients. G J Health Sci.

[24] Tang YL, Zeng JL (2020). Curative effect of Percutaneous Acupoint Electrical Stimulation on Coagulation and blood rheology indexes after knee arthroplasty. Chin J Trad Med Traum & Orthop.

[25] Chen Y, Yang NZ, Hao HM (2022). Study on the level changes of fibrin degradation products, D-dimer, antiprothrombin-III and blood coagulation in pregnant women at different pregnancy periods and their clinical significance. Jilin Med.

[26] Yuan ZF, Yin H, Ma WP (2016). The combined effect of administration of intravenous and topical tranexamic acid on blood loss and transfusion rate in total knee arthroplasty: combined tranexamic acid for TKA. Bone Joint Res.

[27] Liu ZG, Zhang C, Song GR (2020). Efficacy of tranexamic acid on reducing perioperative blood loss during total knee arthroplasty in obese knee osteoarthritis patients. J Chin Pract Diagn Ther.

